# Behandlung einer Aufmerksamkeitsdefizit-/Hyperaktivitätsstörung (ADHS) im höheren Lebensalter – Für ADHS ist es nie zu spät

**DOI:** 10.1007/s00115-025-01807-9

**Published:** 2025-03-05

**Authors:** Svenja Wudy, Nicole Mauche, Jue Huang, Maria Strauß

**Affiliations:** 1 Klinik und Poliklinik für Psychiatrie und Psychotherapie, Universitätsklinikum Leipzig, AöR, Semmelweisstraße 10, 04103 Leipzig, Deutschland; 2 Medizinische Fakultät, Universität Leipzig, Leipzig, Deutschland

## Hintergrund

Die Aufmerksamkeitsdefizit‑/Hyperaktivitätsstörung (ADHS) ist eine Entwicklungsstörung beginnend im Kindesalter und charakterisiert durch Symptome der Aufmerksamkeitsstörung, Impulsivität und motorischen Hyperaktivität [5].

Einige oder alle dieser Symptome bleiben in etwa 40–60 % der Fälle bis ins Erwachsenenalter bestehen [4], was zu einer weltweiten Prävalenz von 5,29 % führt [10]. Inzwischen ist bekannt, dass sich Symptome der Hyperaktivität und Impulsivität über die Lebensspanne verändern und eine geringere Ausprägung zeigen [14]. Studien indizieren eine Prävalenz von ca. 1,5–2,2 % im höheren Lebensalter [3].

Die ADHS im höheren Lebensalter bedingt Besonderheiten in der Diagnostik und im Behandlungsplan. Dazu zählen die Abgrenzung zu anderen psychischen Störung und das erhöhte Risiko für neurodegenerative Erkrankungen. Zudem ist bei der Therapie das Einbeziehen kardiovaskulärer Risikofaktoren erforderlich [12, 13].

Im Folgenden soll das klinische Vorgehen bei der Verdachtsdiagnose ADHS bei älteren Betroffenen anhand zweier Fallbeispiele mit Fokus auf potenzielle Fallstricke beleuchtet werden.

## Fall 1

Berichtet wird von einer 62-jährigen Patientin, die mit einer schweren depressiven Episode bei vorbekannter rezidivierender depressiver Störung und einer bisherigen Therapieresistenz auf eine Spezialstation für affektive Störungen aufgenommen wurde. Die Erstdiagnose Depression wurde vor 24 Jahren gestellt. An somatischen Komorbiditäten lagen eine arterielle Hypertonie sowie Hyperlipidämie vor.

Anamnestisch berichtete sie von mehreren stationären psychiatrischen, psychotherapeutischen und medikamentösen Therapieversuchen, u. a. mit Bupropion, Venlafaxin, Mirtazapin, Amitriptylin und Lithium. Klinisch fielen neben einem depressiven Syndrom, ein desorganisiertes Verhalten, eine erhöhte Ablenkbarkeit mit Störung des zielgerichteten Handelns und ein schlechtes Zeitmanagement auf. Die Patientin berichtete zudem von einer unabhängig von der depressiven Symptomatik vorhandenen inneren Unruhe, welche zu einer Vermeidung langfristiger Ruhe führte.

Im Rahmen der Eingangsdiagnostik für therapieresistente Depressionen erfolgte auch ein ADHS-Screening, das einen positiven Befund ergab. Die ausführliche ADHS-Diagnostik bestätigte den Verdacht (Tab. 1). Eine cMRT-Bildgebung zeigte neben mikroangiopathischen Marklagerläsionen einen altersentsprechenden Befund.

Bei vordergründiger depressiver Symptomatik erfolgte eine Hierarchisierung der Behandlung. Neben einer antidepressiven Kombinationstherapie, bestehend aus Duloxetin 60 mg und Agomelatin 25 mg wurde die Patientin in das multimodale stationäre Behandlungskonzept integriert. Das Duloxetin wurde aufgrund der Präferenz der Patientin eingesetzt, da sie in der Vergangenheit mit diesem Präparat die besten Erfahrungen gemacht hatte. Eine RR- oder Pulserhöhung traten als Nebenwirkung nicht auf. Nach Besserung der depressiven Symptome wurde eine ADHS-spezifische Medikation mit retardiertem Methylphenidat 20 mg begonnen. Leider entwickelte sich hierunter ein Arzneimittelexanthem, sodass ein Wechsel zu Lisdexamphetamin 50 mg erfolgte. Aufgrund des Patientenalters erfolgte die Titration in kleinen Schritten und unter engmaschigem Monitoring der Psychopathologie als auch regelmäßiger Herz- und Kreislaufkontrollen. Aufgrund der bestehenden Hyperlipidämie wurde eine Statintherapie eingeleitet.

Darunter kam es zu einer deutlichen Besserung der depressiven Symptomatik als auch der ADHS-spezifischen Funktionseinschränkungen, sodass die Entlassung ins ambulante Setting erfolgen konnte.

## Fall 2

Ferner wird von einer 65-jährigen Patientin berichtet, die sich ebenso mit einem schweren depressiven Syndrom bei rezidivierender depressiver Störung mit Therapieresistenz zur stationären Aufnahme vorstellte. Die Erstdiagnose Depression wurde vor 23 Jahren gestellt.

Psychopathologisch fiel zusätzlich eine ausgeprägte Aufmerksamkeitsstörung mit desorganisiertem Verhalten auf. Weiterhin zeigten sich in der klinischen Verhaltensbeobachtung der Patientin Probleme, planvoll und effizient zu handeln. Die Patientin berichtete über Probleme der Emotionsregulation und über Schwierigkeiten, spontanen Handelsimpulsen zu widerstehen und Störreize auszublenden. Sie gab an, unter einer inneren Unruhe und Schlafstörungen zu leiden. Sie berichtete, genannte Symptome seit der Kindheit zu kennen. Während depressiver Episoden könne sie diese Probleme schwerer kontrollieren und ausgleichen. An Komorbiditäten ließ sich eine arterielle Hypertonie eruieren.

Auch bei dieser Patientin war das durchgeführte ADHS-Screening positiv, die ausführliche ADHS-Diagnostik bestätigte die Diagnose ADHS (Tab. 1).

Zunächst wurde die antidepressive Medikation aufgrund von Wirkungslosigkeit von Duloxetin 60 mg auf Mirtazapin 15 mg umgestellt.

Aufgrund einer hypertensiven Blutdrucklage erfolgte eine antihypertensive Behandlung mit Amlodipin und Ramipril. Eine kardiologische Vorstellung inklusive einer Langzeitblutdruckmessung ergab keine Kontraindikation für eine medikamentöse Behandlung. Nach Erreichen normotensiver Blutdruckwerte wurde aufgrund des Leidensdrucks der Patientin mit Lisdexamphetamin 30 mg begonnen. Die Blutdruckkontrollen zeigten darunter erneut hypertensive Werte. Außerdem beklagte die Patientin eine Zunahme der Unruhe. Daher erfolgte die Umstellung auf retardiertes Methylphenidat, das langsam auf eine Tagesdosis von 40 mg titriert wurde. Auch hierunter entwickelte die Patientin hypertensive Werte. Zusätzlich beklagte sie Herzrasen und eine anhaltende Diarrhö, weshalb die erneute Umstellung auf Atomoxetin 40 mg bei guter Verträglichkeit erfolgte. Hierunter kam es zu einer deutlichen Besserung sowohl der depressiven als auch der ADHS-Symptome einschließlich der Schlafstörung.

**Tab. 1:** Auszug der standardisierten ADHS-Diagnostik

	Fall 1	Fall 2
*Adult* *ADHD Self-Report Scale*	7/18	16/18
*Wender Utah Rating Scale*	RW 39; KW 7	RW 55; KW 2
*Conners’ Adult ADHD Rating Scale*	Inkonsistenzindex 6 ADHS-Index RW 24 (T = 88) DSM-Unaufmerksamkeit RW 18 (T = 89) DSM-Hyperaktivität/Impulsivität RW 16 (T = 82) DSM-Gesamt RW 34 (T = 90)	Inkonsistenzindex 5 ADHS-Index RW 24 (T = 90) DSM-Unaufmerksamkeit RW 21 (T = 87) DSM-Hyperaktivität/Impulsivität RW 20 (T = 76) DSM-Gesamt RW 41 (T = 90)
*Wender-Reimherr-Interview*	Aufmerksamkeit 2/4 Überaktivität 3/4 Temperament 2/4 Affektive Labilität 1/4 Emotionale Überreagibilität 3/4 Desorganisation 2/4 Impulsivität 2/4 Gesamt: 15/28	Aufmerksamkeit 3/4 Überaktivität 3/4 Temperament 3/4 Affektive Labilität 3/4 Emotionale Überreagibilität 2/4 Desorganisation 3/4 Impulsivität 2/4 Gesamt: 19/28
*Strukturiertes klinisches Interview für DSM-5®-Störungen*	F90.0 F33.2	F90.0 F33.2
*ADHS-Selbstbeurteilungsbogen*	Gesamt RW: 34/54 Unaufmerksamkeit RW 19/27 Hyperaktivität RW 12/15 Impulsivität RW 5/12 DSM-IV Unaufmerksamkeit 9/9 Hyperaktiv/impulsiv 8/9 DSM-IV streng Unaufmerksamkeit 7/9 Hyperaktiv/impulsiv streng 5/9	Gesamt RW: 41/54 Unaufmerksamkeit RW 18/27 Hyperaktivität RW 13/15 Impulsivität RW 10/12 DSM-IV Unaufmerksamkeit 9/9 Hyperaktiv/impulsiv 9/9 DSM-IV streng Unaufmerksamkeit 7/9 Hyperaktiv/impulsiv streng 9/9
*Kölner ADHS-Test für Erwachsene*	RW 144	RW 120

*RW* Rohwert, *KW* Kontrollwert

## Diskussion

Eine ADHS kann auch im höheren Alter als Komorbidität bei affektiven Störungen bestehen. In diesem Zusammenhang ist die ADHS insbesondere bei Therapieresistenz oder bei rezidivierenden depressiven Störungen in die Diagnostik miteinzubeziehen [6].

Auch bei älteren Betroffenen kann eine zuverlässige ADHS-Diagnosestellung erfolgen, wobei das diagnostische Verfahren den S3-Leitlinien für Erwachsene entsprechen sollte. Neben den *Homburger ADHS-Skalen*, welche auch für Erwachsene über 60 Jahren validiert wurden [9], hat sich die *Kurzfassung der Conners’ Adult ADHD Rating Scales* bewährt [2]. Eine Fremdanamnese und beispielsweise Verhaltensbeschreibungen in Grundschulzeugnissen können weitere wertvolle Hinweise liefern.

Das Management somatischer Komorbiditäten ist in dieser Patientengruppe besonders herausfordernd. Auch sollte in den differenzialdiagnostischen Erwägungen das altersbedingt erhöhte Risiko für neurodegenerative Erkrankungen Berücksichtigung finden. Eine umfassende neurologisch-internistische Untersuchung, einschließlich laborchemischer Bestimmung potenzieller Risikoprofile, Herz-Kreislauf-Untersuchungen wie Langzeitblutdruckprofile oder das Hinzuziehen anderer Fachdisziplinen, sollten vor einer medikamentösen Behandlung mit Psychostimulanzien erfolgen [11].

Bei der Indikationsstellung zur Pharmakotherapie älterer Erwachsener mit ADHS sollten Faktoren wie der Schweregrad der Symptomatik und die Beeinträchtigung in verschiedenen Lebensbereichen berücksichtigt werden. Die Präferenzen Betroffener, psychosoziale, psychoedukative und psychotherapeutische Maßnahmen als auch das retrospektive Verständnis eigener Verhaltensmuster ermöglichen einen individualisierten Behandlungsplan und sollten in einem multimodalen Therapiekonzept Berücksichtigung finden. Bereits die Selbsterkenntnis durch die ADHS-Diagnosestellung bietet wertvolle Einblicke in die biographische Entwicklung [1]. Eine reine nichtmedikamentöse Therapie kann vor dem Hintergrund erhöhter Nebenwirkungen bei gleichzeitig fehlendem zielgerichtetem Wissen der ADHS-Behandlung im höheren Lebensalter diskutiert werden.

Obgleich bei älteren Betroffenen mit ADHS spezifische Besonderheiten zu berücksichtigen sind, sollte nach sorgfältiger Abwägung potenzieller Kontraindikationen sowie nach Minimierung entsprechender kardiovaskulärer Risikofaktoren als auch dem adäquaten Rückgang der depressiven Symptomlast eine leitlinienkonforme medikamentöse Behandlung mit Psychostimulanzien in Erwägung gezogen werden. Auch außerhalb des altersgemäßen Zulassungsbereiches einiger Psychostimulanzien kann im Einzelfall eine medikamentöse Behandlung mit Betroffenen diskutiert werden, eine entsprechende Aufklärung über das unter Psychostimulanzien depressiogene sowie kardiovaskuläre Nebenwirkungspotenzial sowie die Nutzung des Medikaments im Off-label-Bereich sollte erfolgen.

Entgegen des beschriebenen depressiogenen Nebenwirkungspotenzials konnten Lavretsky et al. sogar eine signifikante Überlegenheit bei gleichzeitig guter Verträglichkeit einer Kombinationstherapie bestehend aus Citalopram sowie Methylphenidat im Vergleich zu Citalopram plus Placebo oder Methylphenidat plus Placebo bei der medikamentösen Behandlung älterer depressiver Patienten aufzeigen [7]. Eine Metaanalyse aus dem Jahr 2022 zeigte ebenfalls eine gute kardiovaskuläre Verträglichkeit ADHS-spezifischer Medikation, einschließlich Psychostimulanzien [8]. Dennoch konnte auch im Rahmen einer Kohortenstudie bei älteren Erwachsenen ein erhöhtes Risiko für kardiovaskuläre Ereignisse durch die Einnahme von Stimulanzien, insbesondere bei Therapiebeginn dargestellt werden, während für die Langzeiteinnahme kein Zusammenhang bestand [12].

## Fazit für die Praxis

- Das Vorliegen einer ADHS im höheren Lebensalter ist nicht ausgeschlossen und sollte leitliniengerecht diagnostiziert und behandelt werden.
- Auch bei dieser Altersgruppe kann eine verlässliche ADHS-Diagnose gestellt werden.
- Die Indikation für eine Behandlung mit Psychostimulanzien sollte nach sorgfältiger Abwägung erfolgen.
- Somatische Erkrankungen sollten in den Behandlungsplan integriert werden, eine interdisziplinäre Zusammenarbeit ist hierbei besonders empfehlenswert.
